# Responses of nitrogen efficiency and antioxidant system of summer maize to waterlogging stress under different tillage

**DOI:** 10.7717/peerj.11834

**Published:** 2021-07-26

**Authors:** Baizhao Ren, Juan Hu, Peng Liu, Bin Zhao, Jiwang Zhang

**Affiliations:** Shandong Agricultural University, Taian, China

**Keywords:** Ridge tillage, Summer maize, Waterlogging, Nitrogen efficiency

## Abstract

Waterlogging was one of the main abiotic stresses affecting maize yield and growth in the North China Plain, while ridge tillage effectually improved soil environment, enhanced crop stress resistance to waterlogging, and increased grain yield of waterlogged maize. In order to explore the responses of nitrogen (N) efficiency and antioxidant system of summer maize to waterlogging stress under different tillage, a field experiment was conducted to explore N use efficiency, leaf activities of superoxide dismutase (SOD), peroxidase (POD), and catalase (CAT), and malondialdehyde (MDA) content of waterlogged maize Denghai 605 (DH605) and Zhengdan 958 (ZD958) under different tillage system (ridge planting and flat planting). Our results showed that ridge tillage was beneficial to ameliorate waterlogging damages on antioxidant system by increasing SOD, POD, and CAT activities, and decreasing MDA content. Moreover, ridge tillage significantly increased N efficiency of waterlogged maize. N translocation amount (NTA), N translocation efficiency (NTE), N contribution proportion (NCP), N harvest index (NHI), and N use efficiency (NUE) of waterlogging treatment under ridge planting system (W-V3+R) for DH605 was increased by 108%, 69%, 60%, 8% and 16%, while ZD958 increased by 248%, 132%, 146%, 13% and 16%, respectively, compared to those of waterlogging treatment under flat planting system (W-V3). Ultimately, ridge tillage led to a significant yield improvement by 39% and 50% for DH605 and ZD958, respectively, compared to that of W-V3. In conclusion, ridge tillage was conducive to retard leaf aging, and enhance nitrogen efficiency, thereby resulting in a yield improvement of waterlogged summer maize.

## Introduction

With global climate change, the frequency and intensity of extreme precipitation events is increased (Bandh et al., 2021). Waterlogging become one of the main abiotic stresses affecting crop growth and development (Rajkhowa & Sarma, 2021). One-tenth of all irrigation fields is reckoned to be affected by unseasonal and serious flooding, causing crop losses by as much as 20% (Laurentius & Julia, 2015). The disaster area of the Yangtze River Basin and the North China Plain accounts for about 75% of the total disaster area in China (Yu & Zhao, 2013). In the North China Plain, summer maize is unseasonably subjected to waterlogging stress, because most rainfall concentrated in growth periods of summer maize. In recent years, waterlogging occurred frequently at the seedling and flower period of summer maize, and thus affecting maize yield and growth (Ren et al., 2014). The decrease of grain yield after waterlogging may be due to the deficit of soil oxygen, and the inhibition of root ability (Kaur et al., 2020), which leads to changes in the composition and decomposition activities of microbes, the disorder of nutrient recycling process, and the reductions of nutrient absorption and accumulation (Tian et al., 2021). After a prolonged period of waterlogging stress, leaf normal carbon metabolism and nitrogen (N) metabolism is inhibited (Vwioko, Adinkwu & El-Esawi, 2017; Ren et al., 2018), contributing to the degenerated photo-system, and the reduction of N efficiency, and thus limiting physiological processes associated with N, results in a significant yield decrease of summer maize (Vwioko, Adinkwu & El-Esawi, 2017; Ren et al., 2018). Moreover, waterlogging leads to the destruction of antioxidant system, and the increase of malondialdehyde (MDA) content, indicating the detrimental effects of waterlogging on membrane lipid peroxidation and integrity, and thus accelerating leaf aging (Pan et al., 2021), results in the disturbance of leaf physiological functions, eventually inhibiting dry matter accumulation and yield formation of crops (Ren et al., 2018; Zhang et al., 2021).

Waterlogging mainly happened in areas with high groundwater level, poor drainage and ventilation (Kaur et al., 2020). Under flat planting, waterlogging significantly damages maize growth, leading to a significant yield reduction (Ren et al., 2018). However, ridge tillage is conducive to improve soil environment, and enhance crop stress resistance, eventually leading to the increase of crop yield (Abagandura, Mohamed Nasr & Moumen, 2017; Dong et al., 2017). The planting mode of ridge cultivation can be adopted to easily waterlogged areas (Li et al., 2018). Broad-row ridge cultivation effectively improve photosynthetic characteristics and lodging resistance, and increase grain yield of summer maize with waterlogging at seedlings stage (Wang et al., 2015). Our previous studies also showed that ridge tillage was beneficial to improve canopy structure and photosynthetic characteristics of waterlogged summer maize, ultimately ameliorating waterlogging damages on maize yield and growth (Ren et al., 2016). Nitrogen (N) is closely related to crop growth and organ formation (Kitonyo et al., 2018). Improving N use efficiency is ome of important ways to improve yield and quality of crops (Liu et al., 2021). The lack of N would lead to the breakdown of soluble protein and the increase of malondialdehyde (MDA) content, which damages protective enzyme system, and thus accelerating leaf aging (Li et al., 2021). However, ridage tillage is conducive to enhance root vitality, and improve absorption capacity (Abagandura, Mohamed Nasr & Moumen, 2017; Qin et al., 2020), which contributes to the absorption and assimilation of nutrient, and thus improving crop yield and quality.

Visibly, ridge tillage can effectually improve N efficiency and antioxidant system, and enhance crop resistance to abiotic stresses. However, very little attention has been given to assess the physiological mechanism of ridge planting to regulate yield and growth of waterlogged maize from the aspects of N efficiency and antioxidant system. In this study, we performed a field experiment to explore waterlogging stresses on antioxidant enzymes activities, MDA content, N accumulation and distribution, and N use efficiency of summer maize under different planting system, which will illuminate the regulation mechanism of ridge tillage to antioxidative system and N efficiency of waterlogged summer maize.

## Materials and Methods

### Plant materials and experimental design

A field experiment was conducted at the experimental farm (36°10′N, 117°04′E, 151 m a.s.l.) maintained by the State Key Laboratory of Crop Biology of Shandong Agricultural University, China in 2014 and 2015. This area belongs to a temperate continental monsoon climate. The experimental topsoil (0–20 cm) type is brown loam with 10.7 g kg^−1^ organic matter, 0.9 g kg^−1^ total N, 50.7 mg kg^−1^ rapidly available P, and 86.2 mg kg^−1^ rapidly available K before the experiment. The effective accumulated temperature was 1,740.9 °C d and 1,710.5 °C d, and the mean total precipitation was 356.0 and 378.5 mm during the growth periods of summer maize in 2014 and 2015, respectively. Summer maize hybrids Denghai605 (DH605) and Zhengdan958 (ZD958), the most widely planted hybrids in China, were used as experimental materials. Maize was sown on June 16 at 67,500 plants ha^−1^ in both experimental years. Experimental treatments were as follows: tillage treatments (ridge planting, and flat planting), and water treatments (waterlogging, and no waterlogging). Details of experimental treatments (Table 1), according to our previously described procedure (Ren et al., 2016). There was a separate water supply pipe in each waterlgging plot. The water level above soil surface was maintained at 2-3 cm for 6 days by controlling water discharge with water valve. Disease, weeds, and pests were well-controlled in each treatment. Each treatment was replicated three times, in a completely randomized block design.

**Table 1:** Experimental treatments in the field. Ridge tillage was with ridge scope of 120 cm, ridge surface width of 100 cm, ridge ditch width of 20 cm, and ridge height of 15 cm (Fig. 1). Summer maize was sowed in ridge surface, with row spacing of 60 cm and plant spacing of 24.5 cm, at a plant density of 67,500 plants ha^−1^; Conventional tillage was conventional flat planting with row spacing of 60 cm and plant spacing of 24.5 cm, at a plant density of 67,500 plants ha^−1^.

Water treatment	Planting pattern	Abbreviation
Waterlogging at the third leaf stage for 6 days	Conventional tillage	W-V3
	Ridge tillage	W-V3+R
No waterlogging	Conventional tillage	CK

### Nitrogen efficiency

At the sixth leaf stage (V6), the twelfth leaf stage (V12), the tasseling stage (VT), the milk stage (R3), and the physiological maturity stage (R6), according to Ritchie & Hanway (1982), the five representative plant samples were obtained from each plot to calculate nitrogen (N) content. Samples were preserved after being separated into stem and leaf at V6, V12 and VT, and separated into stem, leaf and ear at R3 and R6. Samples were dried at 80 °C in a force-draft oven (DHG-9420A; Bilon Instruments Co., Ltd, Shanghai, China) to a constant weight, and weighed separately. After weighing, the samples were grounded using a cyclone sample mill with a fine mesh (0.5 mm), and then the N concentrations of different organs were measured with the micro-Kjeldahl method (CN61M/KDY-9820; Beijing, China) (Li et al., 2017). Furthermore, N use efficiency (NUE), N harvest index (NHI), N translocation amount (NTA), N translocation efficiency (NTE) and N contribution proportion (NCP) were calculated, according to the following equations (Li et al., 2017; Ren et al., 2017):

NUE (kg kg^–1^) = grain yield/total N uptake amount by plant

NHI (%) = grain N amount/total N amount of plant

NTA (g/plant) = total N amount at VT – total N amount excluding grain N amount at R6

NTE (%) = NTA/ total N amount at VT

NCP (%) = grain N amount/NTA

### Antioxidant enzymes activities and malondialdehyde (MDA) content

At V6, V12, VT, R3 and R6, the functional leaves from three plant samples were obtained to analyze the activities of Superoxide dismutase (SOD), Peroxidase (POD), and Catalase (CAT), and Malondialdehyde (MDA) content, according to the method we described previously (Ren et al., 2018).

### Yield

At R6, 30 ears were harvested from three rows at the center of each plot to analysis yield, and yield components. Each treatment was repeated three times

### Statistical analysis

Analysis of variance (ANOVA) was performed according to the general linear model procedure of SPSS (Ver. 17.0; SPSS, Chicago, IL, USA). The least significant difference (LSD) between the means was estimated at the 95% confidence level.

## Results

### Leaf antioxidative enzyme activities

**Figure 1:**
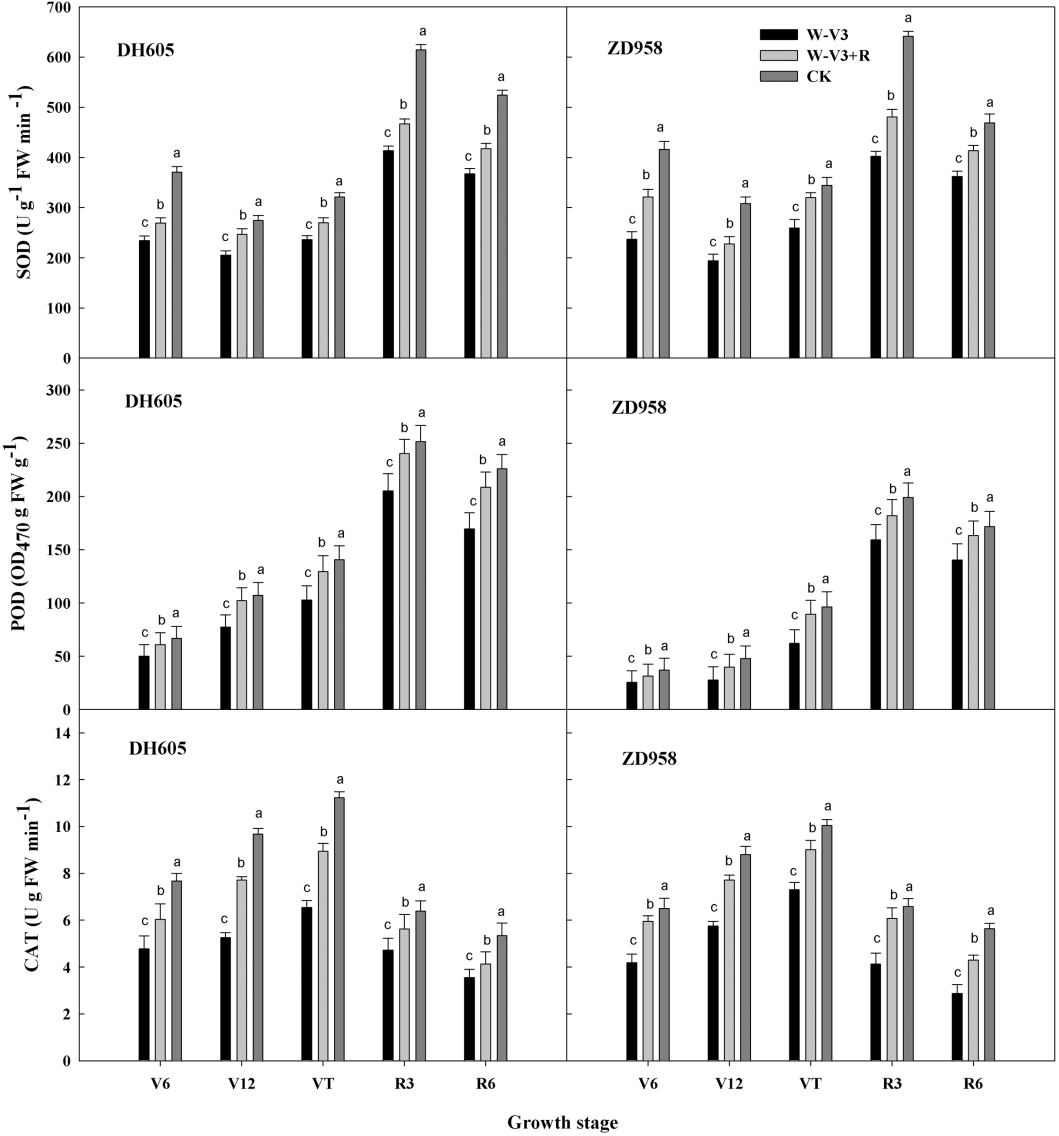
Ridge tillage increased the activity of leaf protective enzyme of waterlogged summer maize. W-V3: waterlogging at the third leaf stage under conventional tillage; W-V3+R: waterlogging at the third leaf stage under ridge tillage; CK: no waterlogging under conventional tillage. SOD: superoxide dismutase; POD: peroxidase; CAT: catalase. V6: the sixth leaf stage; V12: the twelfth leaf stage; VT: the tasseling stage; R3: the milk stage; R6: the physiological maturity stage. Means and standard errors based on three replicates are shown. Values followed by a different small letter for each development stage are significantly different at 5% probability level (*p* < 0.05).

The activities of leaf antioxidative enzyme were significantly changed after waterlogging. The activities of superoxide dismutase (SOD), peroxidase (POD), and catalase (CAT) for waterlogging treatments were lower than those in the CK treatment at the corresponding growth stages. The overall trend of SOD, POD and CAT activities was consistent among the treatments. Overall, the activities increased firstly and then decreased at growth period. The activities of SOD, POD and CAT of W-V3 (waterlogging treatment under flat planting) for DH605 were averagely significantly decreased by 30%, 25% and 37% for each developmental stage, respectively, compared to those of CK. Those of W-V3 for ZD958 were averagely decreased by 33%, 29% and 37% respectively, compared to those of CK. However, ridge tillage effectually mitigated waterlogging stress on antioxidative enzyme activities. The activities of SOD, POD, and CAT for DH605 under W-V3+R (waterlogging treatment under ridge tillage) were averagely significantly increased by 15%, 24% and 29% for each developmental stage, respectively, compared to those under W-V3. Those of ZD958 under W-V3+R were averagely increased by 22%, 28% and 39% respectively, compared to those under W-V3 (Fig. 1).

**Figure 2:**
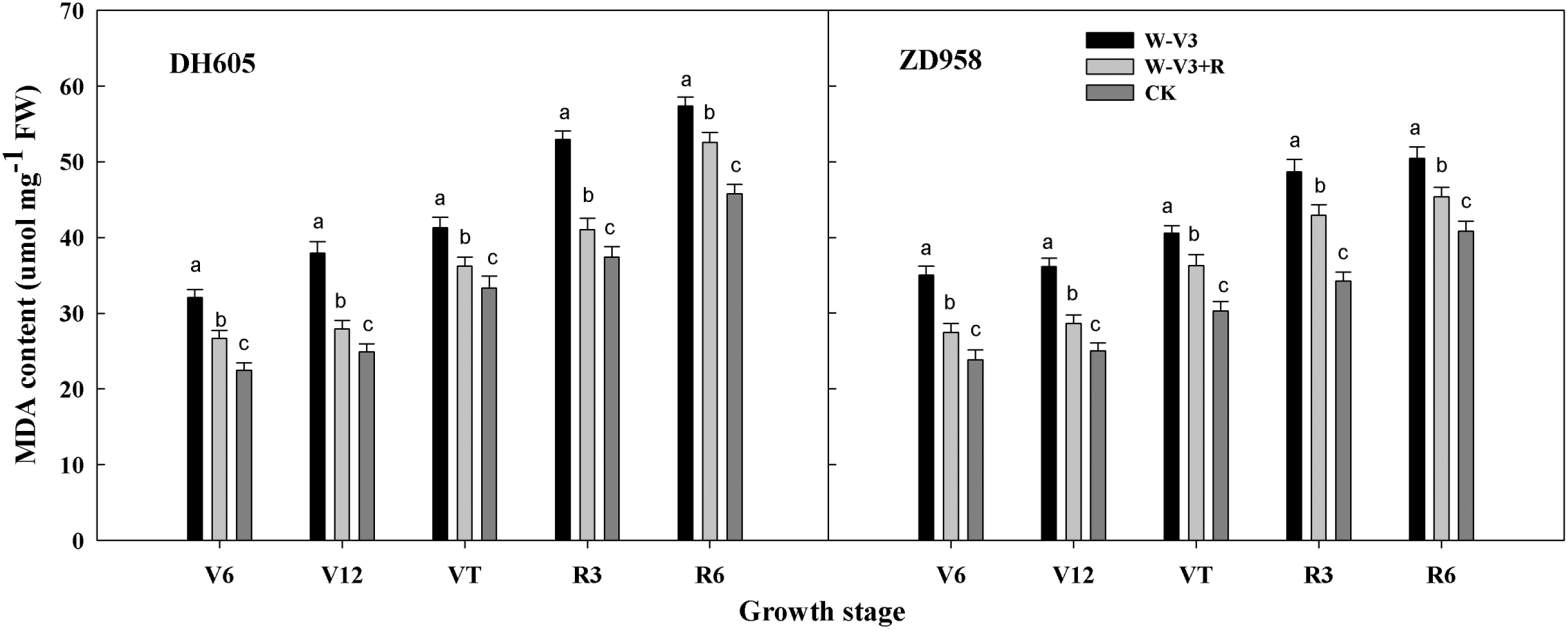
Ridge tillage lessened leaf malondialdehyde (MDA) content of waterlogged summer maize. W-V3: waterlogging at the third leaf stage under conventional tillage; W-V3+R: waterlogging at the third leaf stage under ridge tillage; CK: no waterlogging under conventional tillage. V6: the sixth leaf stage; V12: the twelfth leaf stage; VT: the tasseling stage; R3: the milk stage; R6: the physiological maturity stage. Means and standard errors based on three replicates are shown. Values followed by a different small letter for each development stage are significantly different at 5% probability level (*p* < 0.05).

In addition, the malondialdehyde (MDA) content was significantly increased after waterlogging at the corresponding growth stages, compared to CK. However, ridge tillage was beneficial to alleviate the increase of MDA content induced by waterlogging. The MDA content of W-V3+R for DH605 and ZD958 was averagely decreased by 17% and 15% for each developmental stage, respectively, compared to that of W-V3 (Fig. 2).

### Nitrogen (N) accumulation and distribution

**Figure 3:**
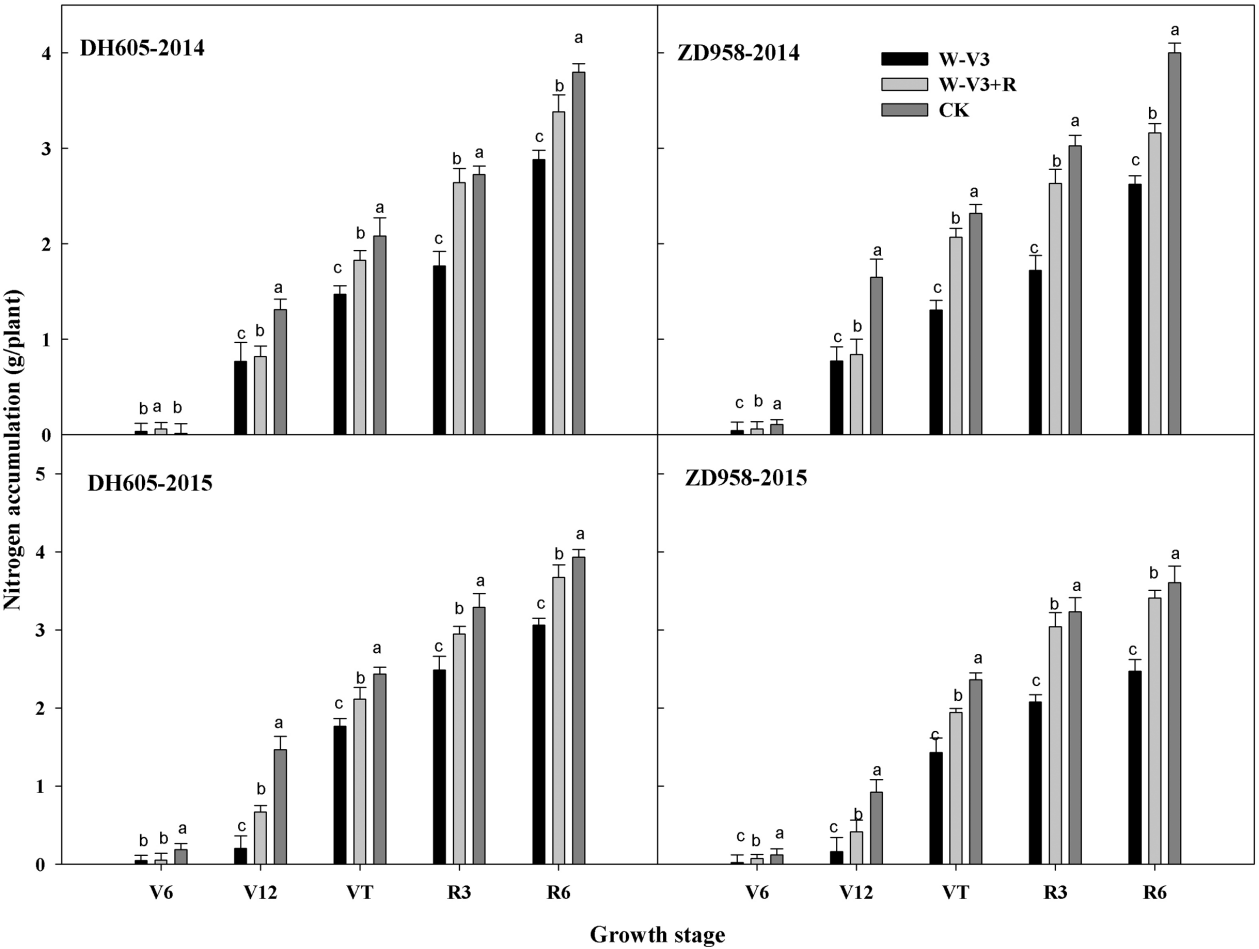
Ridge tillage enhanced N accumulation of waterlogged summer maize at different stage. W-V3: waterlogging at the third leaf stage under conventional tillage; W-V3+R: waterlogging at the third leaf stage under ridge tillage; CK: no waterlogging under conventional tillage. V6: the sixth leaf stage; V12: the twelfth leaf stage; VT: the tasseling stage; R3: the milk stage; R6: the physiological maturity stage. Means and standard errors based on three replicates are shown. Values followed by a different small letter for each development stage are significantly different at 5% probability level (*p* < 0.05).

**Figure 4:**
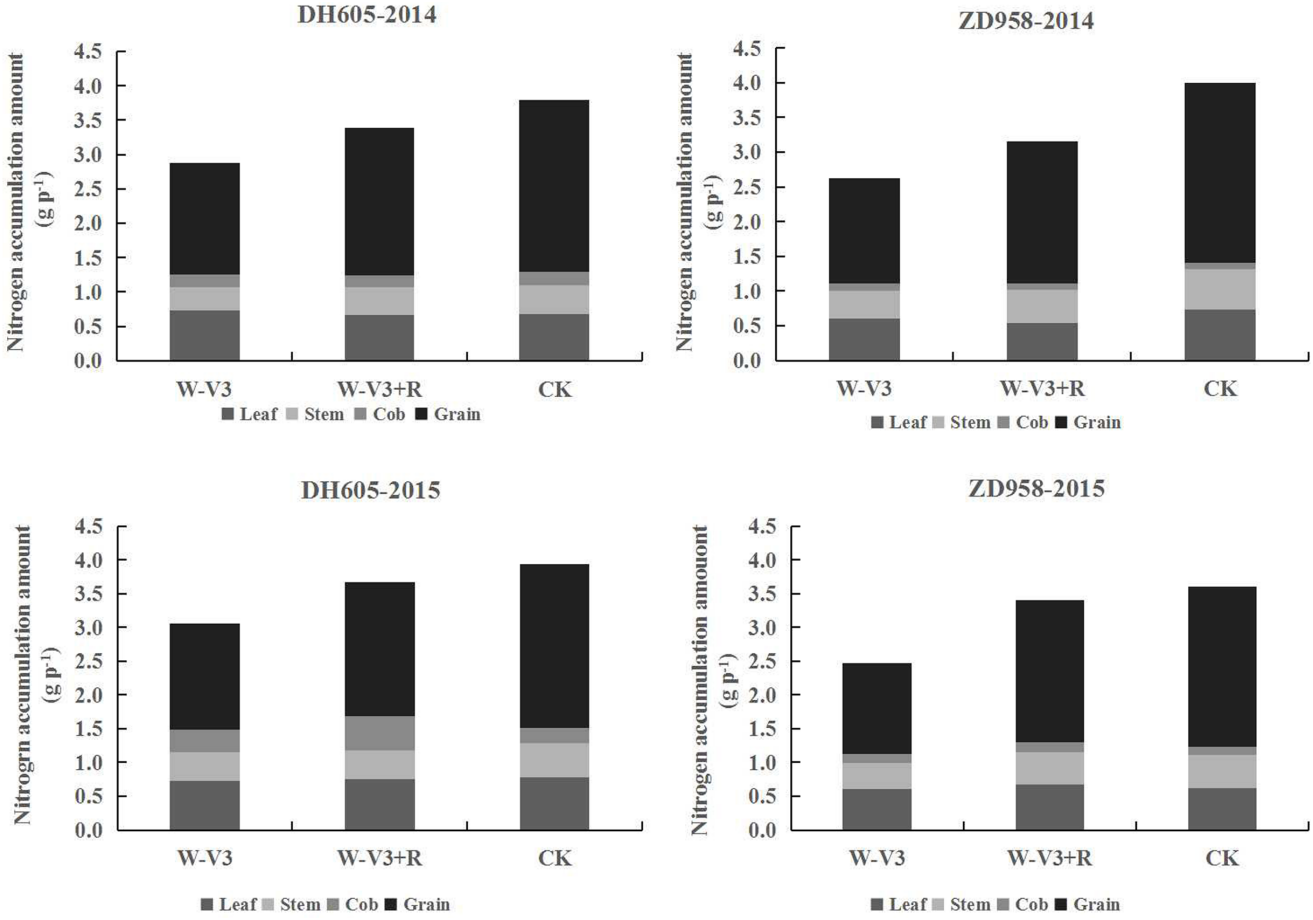
Ridge tillage improved N accumulation and translocation of waterlogged summer maize at the physiological maturity stage. W-V3: waterlogging at the third leaf stage under conventional tillage; W-V3+R: waterlogging at the third leaf stage under ridge tillage; CK: no waterlogging under conventional tillage.

The N accumulation of DH605 and ZD958 were averagely reduced after waterlogging by 23% and 33% for each developmental stage across years, respectively, compared to that of CK. Following waterlogging stress, there was an improved N accumulation of waterlogged summer maize under the ridge tillage at each stage. N accumulation of W-V3+R for DH605 and ZD958 was improved by 18% and 29% on the average, respectively, compared to that of W-V3 (Fig. 3). Furthermore, waterlogging significantly affected N accumulation and distribution of each organ. Grain N content of W-V3 for DH605 and ZD958 was averagely decreased by 35% and 43% for each developmental stage across years, compared to those of CK. However, ridge tillage effectively improved N accumulation and distribution of each organ by 29% and 46% on the average, respectively, for each developmental stage across years, compared to those of W-V3 (Fig. 4).

### Nitrogen (N) efficiency and grain yield

The N efficiency of summer maize was obviously declined due to waterlogging. N translocation amount (NTA), N translocation efficiency (NTE), N contribution proportion (NCP), N harvest index (NHI), and N use efficiency (NUE) of W-V3 for DH605 were 76%, 64%, 63%, 15% and 20% lower, while those of ZD958 were reduced by 79%, 62%, 64%, 14% and 14% across years, respectively, compared to those of CK. However, ridge tillage helped to improve N efficiency of waterlogged maize. NTA, NTE, NCP, NHI, and NUE of W-V3+R for DH605 were 108%, 69%, 60%, 8% and 16% higher than those of W-V3, respectively. Those of W-V3+R for ZD958 were enhanced by 248%, 132%, 146%, 13% and 16% across years, respectively, compared to those of W-V3 (Table 2). The reduction of N efficiency after waterlogging led to yield decrease by 38% and 42% for DH605 and ZD958 across years, respectively, compared to that of CK. However, ridge tillage effectually increased grain yield by 38% and 50% for DH605 and ZD958 across years, respectively, compared to that of W-V3 (Table 2).

**Table 2:** Effects of ridge tillage on grain yield and N use efficiency of waterlogged summer maize.

Year	Hybrid	Treatment	Grain yield (Kg/ha)	Nitrogen translocation amount, NTA	Nitrogen translocation efficiency (NTE, %)	Nitrogen contribution proportion (NCP, %)	Nitrogen harvest index (NHI)	Nitrogen use efficiency (NUE, %)
				(g/plant)				
2014	DH605	W-V3	9,310 c	0.22 c	14.97 c	13.50 c	0.57 b	47.89 c
		W-V3+R	11,495 b	0.59 b	32.24 b	27.44 b	0.63 a	50.23 b
		CK	14,336 a	1.29 a	50.00 a	51.39 a	0.66 a	55.89 a
	ZD958	W-V3	8,093 c	0.20 c	15.27 b	13.25 c	0.58 b	45.76 b
		W-V3+R	11,306 b	0.96 b	46.38 a	46.83 b	0.65 a	53.01 a
		CK	13,916 a	1.31 a	48.16 a	50.58 a	0.65 a	51.54 a
2015	DH605	W-V3	8,311 c	0.29 c	16.33 c	18.28 c	0.52 b	40.24 b
		W-V3+R	12,646 b	0.43 b	20.18 b	21.51 b	0.54 b	51.01 a
		CK	14,175 a	0.92 a	37.96 a	38.18 a	0.62 a	53.44 a
	ZD958	W-V3	8,137 c	0.30 b	20.81 c	22.16 c	0.54 c	48.81 b
		W-V3+R	13,107 b	0.64 b	33.14 b	30.57 b	0.62 b	57.01 a
		CK	14,164 a	1.13 a	47.66 a	47.51 a	0.66 a	58.19 a

**Note:**

W-V3: waterlogging at the third leaf stage under conventional tillage; W-V3+R: waterlogging at the third leaf stage under ridge tillage; CK: no waterlogging under conventional tillage. Values followed by a different small letter within a column are significantly different at 5% probability level. Differences between treatments were calculated within the hybrids for each particular year.

## Discussion

Waterlogging led to soil oxygen deficiency, resulting in the inhibition of nutrient uptake and transportation, and the decrease of grain yield (Tian et al., 2021). Nitrogen (N) was one of important soil nutrients that affecting crop growth, and organ formation (Kitonyo et al., 2018; Li et al., 2021). N metabolism was a basic physiological process, of which related enzymes played an important role in plant physiological activities (Limami, Diab & Lothier, 2014). Soil water content affected not only soil N availability, but also N absorption, transportation and assimilation during crop growth process (Ruiz et al., 2021). Previous studies showed that leaf N content was significantly declined after waterlogging (Ren et al., 2017), restricting leaf photosynthesis and the activity of RuBPcase enzyme, resulted in the decrease of photosynthetic product (Vwioko, Adinkwu & El-Esawi, 2017), which in turn affected N uptake, and thus resulting in yield reductions. Our study showed that waterlogging significantly decreased N accumulation of each organ, and grain N distribution rate of summer maize, resulting in the decrease of N efficiency, which indicated that waterlogging inhibited maize N absorption, restricting physiological processes associated with N. As a result, plant photosynthesis production and accumulation was weakened by waterlogging, ultimately leading to a significant reduction of maize yield (Vwioko, Adinkwu & El-Esawi, 2017). N nutrients required for crop growth and development were mainly absorbed from soil by root system. Waterlogging restrained root absorption capacity, and thus decreasing the level of N absorption and distribution, eventually led to the inhibition of N metabolism and protein synthesis, and the disorder of C/N metabolism (Vwioko, Adinkwu & El-Esawi, 2017; Ren et al., 2018; Zhang et al., 2021). However, ridge tillage was conducive to improve N accumulation and distribution of waterlogged summer maize (Fig. 4), and enhance N efficiency, thus leading to a significant yield increase of waterlogged summer maize. It was mainly attributed to the ridge cropping, which helped to improve the inhabitable environment of root system, and enhance root vitality and absorption capacity (Xu et al., 2018; Qin et al., 2020). In addition, ridge tillage could make root mainly distribute within the space of ridge platform, leading to the more uniform root distribution in the whole space, and the large volume of capillary root (Dong et al., 2017; Xu et al., 2018), which contributed to improve nutrient transportation and assimilation, and increase the proportion of grain N accumulation and distribution, and then increasing N utilization efficiency and grain yield of waterlogged summer maize.

The inhibition of N uptake and transportation would result in the breakdown of soluble protein and the increase of MDA content, leading to the disorder of reactive oxygen species (ROS) scavenging system, and the acceleration of leaf senescence (Li et al., 2021). ROS scavenging system played an important role in protecting cells from photooxidative damage (Ismail, 2018; Hasanuzzaman et al., 2020). Crops could keep the dynamic equilibrium of ROS by antioxidant enzyme systems, and thus mitigating membrane peroxidation, and decreasing the degree of oxidative damage induced by abiotic stresses (low temperature, waterlogging, drought, etc) (Chen et al., 2019; Hasanuzzaman et al., 2020; Pan et al., 2021). SOD, POD and CAT were mainly antioxidant enzymes of plant cells, of which activities reflected plant anti-aging ability (Ashraf et al., 2018; Farooq et al., 2018). MDA content, an important indicator, reflected the degree of membrane lipid peroxidation. Our study showed that the enzyme activities of SOD, CAT, and POD were decreased, while MDA content was increased after waterlogging, suggesting detrimental waterlogging stress on antioxidant enzyme systems, leadig to the unbalance of ROS, and membrane deterioration, thus expediting leaf aging. ROS-scavenging ability of crops can be improved by increasing the activities of antioxidant enzyme that ensured them to alleviate oxidative damage induced by waterlogging (Pan et al., 2021). Ridge cropping helped to improve soil root activity and delay root senescence, providing sufficient nutrient supply for the aboveground organ, and thus enhanced antioxidant activities (Tong et al., 2018). Our results showed that ridge tillage was beneficial to enhance the activities of SOD, POD and CAT, and decrease MDA content, indicating that ridge tillage effectively enhanced ROS-scavenging ability of waterlogged maize by increasing antioxidant enzyme activities, countering oxidative stress, and alleviated waterlogging damages on cell membrane system, resulted in a relatively stable biological membrane. These results showed that ridge tillage could effectively reduce the damage of waterlogging to leaves, timely removed reactive oxygen species within a certain range, maintaining the function of leaves, and thus improving growth and yield of waterlogged maize.

The results from the present study showed that ridage tillage was a viable approach to improve N efficiency and leaf antioxidative system of waterlogged maize. The potental areas of future study should focus on the regulation mechanism of ridge farming analyzed from the aspects of root growth, and soil chemicophysical properties, etc.

## Conclusions

Ridge tillage was conducive to improve antioxidant enzymes activities and nitrogen accumulation and distribution, and decrease MDA content, and thus enhancing nitrogen efficiency, resulted in yield increase of waterlogged summer maize.

## Supplemental Information

10.7717/peerj.11834/supp-1 Supplemental Information 1 Raw data for tables and figures.

## Additional Information and Declarations

### Data Availability

The following information was supplied regarding data availability: The raw data are available in the Supplemental File.
